# Diet and dog characteristics affect major and trace elements in hair and blood of healthy dogs

**DOI:** 10.1007/s11259-021-09854-8

**Published:** 2021-11-06

**Authors:** Sarah Rosendahl, Johanna Anturaniemi, Kristiina A. Vuori, Robin Moore, Manal Hemida, Anna Hielm-Björkman

**Affiliations:** Faculty of Veterinary Medicine, Department of Equine and Small Animal Medicine, University of Helsinki, Helsinki, Finland

**Keywords:** Major elements, Trace elements, Toxic metals, Hair, Blood, Canine

## Abstract

Obtaining correct amounts of essential elements, and avoiding toxic metals are key factors in dog health. Through analyzing major and trace elements in hair and blood of 50 healthy companion dogs using ICP-MS, we study their associations with dog characteristics and diet, hypothesizing that eating the same diet long-term results in strong correlations between hair and blood element concentrations, and that dog characteristics and diet affect element status. The correlation between hair and blood was significant for Hg (R = 0.601, p = 0.000) and Pb (R = 0.384, p = 0.010). The following associations were significant (p < 0.05): Dark hair had higher Ca and Mg compared to light hair. Females had higher hair Zn, blood Mn, and blood As compared to males. Blood Mn and Se increased, while blood Pb decreased with age. Raw diet fed dogs had higher hair Zn and Se compared to dry or mixed diet fed dogs, and lower blood Mn compared to dry diet fed dogs. Dry and mixed diet fed dogs had higher blood Cd compared to raw diet fed dogs. Mixed diet fed dogs had higher hair Ca and Mg compared to raw or dry diet fed dogs, and higher hair Pb compared to dry diet fed dogs. Wild game consumption was associated with higher blood Pb, and rice consumption with higher blood As. In conclusion, hair provides an alternative for assessing Hg and Pb exposure, and major and trace elements status is affected by hair color, sex, age, and diet.

**Supplementary Information:**

The online version contains supplementary material available at 10.1007/s11259-021-09854-8.

## Introduction

To maintain optimal health, dogs need to obtain the right amounts of major and trace elements, while avoiding exposure to harmful chemicals such as toxic metals. Recent studies have further emphasized the importance of element status in dog health by suggesting that trace elements play a role in the pathogenesis of several canine diseases (Vitale et al. 2019; Cedeño et al. 2020). Major elements, including calcium (Ca), magnesium (Mg), phosphorus (P), sodium (Na), and potassium (K), are needed by the body in larger quantities, whereas trace elements, such as iron (Fe), copper (Cu), zinc (Zn), manganese (Mn), selenium (Se), and chromium (Cr) are needed in smaller quantities. Major elements are involved in bone and teeth formation, nerve and muscle function, cell signaling, and acid-base balance, while trace elements act as co-factors in enzymes, and play important roles in antioxidant, hormone, and immune system functions (National Research Council 2006; Puertollano et al. 2011). Conversely, toxic metals, such as lead (Pb), mercury (Hg), cadmium (Cd), arsenic (As), aluminum (Al), and nickel (Ni) can disrupt neurological, reproductive, renal, and hematological systems. They also interfere with the absorption and metabolism of trace elements and increase oxidative stress (D’Souza et al. 2003).

There have been reports of dry dog foods providing either insufficient, excessive, or an inappropriate balance of major and trace elements such as Ca, P, Zn, Cu, Mn, and Se (Paulelli et al. 2018; Pereira et al. 2018; Kazimierska et al. 2020). Davies et al. (2017a) found that a majority of complete wet and dry pet foods sold in the UK were non-compliant according to current European guidelines. Meanwhile, raw food diets have been found to commonly be low in certain major and trace elements such as Ca, Zn, and Cu (Dillitzer et al. 2011). Excess Ca, or the presence of certain other dietary substances, such as phytate in grains, can also negatively impact the absorption of trace elements from the diet (National Research Council 2006). In addition, the absorption of elements can vary depending on elemental form, i.e. dry dog foods are commonly supplemented with inorganic elements that are less bioavailable compared to organic forms (Trevizan et al. 2013). Raw food diets for dogs contain organic elements from raw ingredients, such as meat, fish, bone, offal, eggs, and vegetables, and they are sometimes supplemented with additional organic, or inorganic elements (National Research Council 2006).

Several authors have also expressed concern about long-term exposure to toxic metals in certain dry dog foods (Davies et al. 2017a; De Nadai Fernandes et al. 2018; Kim et al. 2018; Paulelli et al. 2018; Rosendahl et al. 2020). Studies have shown higher concentrations of toxic metals in serum and liver in dogs that eat commercial diets compared to dogs that eat home-made diets (Lopez-Alonso et al. 2007; Tomza-Marciniak et al. 2012). Moreover, some specific dietary items such as wild game and rice have been associated with toxic metal exposure in dogs (Høgåsen et al. 2016; Rosendahl et al. 2020).

A dog’s major and trace element status can be assessed from various loci such as hair and blood. Hair analysis provides a reading of elemental deposition in the cells and interstitial spaces of the hair over a 2-3-month period, and thus gives a long-term assessment of element status or toxic metal exposure. On the other hand, blood analysis indicates the current intake of elements (Jenkins 1979; Ahmad et al. 2013). As blood is prone to fluctuations and homeostatic regulation, hair has been considered a more stable medium for reflecting dietary intake of elements in humans and other animals (Perry et al. 1976; Ghorbani et al. 2015; Kim et al. 2016). During the last decade the interest in hair analysis among canine researchers has increased and it has become evident that not only dietary intake, but also age, sex, hair color, physiological status, health status, living environment, laboratory washing procedures, and in some cases breed, may also affect hair element concentrations in dogs (Chyla and Zyrnicki 2000; Park et al. 2005; So et al. 2016; Davies et al. 2017b; Sgorlon et al. 2019; Chun et al. 2020). There is still a paucity of data on major and trace element, and toxic metal concentrations in hair (So et al. 2016; Davies et al. 2017b; Sgorlon et al. 2019) and blood (Panda et al. 2009; Viviano and Vanderwielen 2013; Sousa et al. 2013; Bahovschi et al. 2015; Ferreira et al. 2017; Langlois et al. 2017) of healthy dogs. Furthermore, research on the correlation between hair and blood element concentrations (Sousa et al. 2013), and the effect of common diet types, such as dry and raw food, on element status (Anturaniemi et al. 2020) is scarce.

The purpose of this study was to establish mean concentrations of major and trace elements in hair and blood of clinically healthy companion dogs to decide whether hair and blood element concentrations correlate with each other, and to assess the effect of age, sex, hair color, and diet on these elements. Our hypothesis was that major and trace elements correlate between hair and blood in dogs, since dogs are usually fed with the same diet for long periods of time. We also hypothesized that element status and toxic metal burden varies depending on which diet type the dog is eating, and that age, sex, and hair color affect element concentrations.

## Materials and methods

### Animals and study design

This was a non-controlled, cross-sectional study including a questionnaire and blood and hair samples from companion dogs living in their home-environment in two different regions in Finland, consisting of both urban, sub-urban, and rural environmental areas. Client-owned companion dogs (N = 50) were recruited via local dog groups on Facebook and then called for a visit to the Helsinki University small animal hospital or to a veterinary clinic in Pohjanmaa (Finland) for collection of hair and blood samples. Inclusion criteria were (i) dogs older than 1 year of age and over 10 kg of body weight, and (ii) dogs that had been eating their current diet for a minimum of six months. Exclusion criteria were (i) disease or therapy and (ii) pregnancy or lactation. After the visits, the dog owners filled in an online questionnaire about their dogs’ basic information, health status, and feeding. The health status of the dogs was determined by using owner-reported health information, a clinical examination, a complete blood count, and serum chemistry. The study included a large variety of breeds: thirteen mixed-breeds, four Nova Scotia Duck Tolling Retrievers, three Samoyeds, German Shepherds, and White Shepherds, two Gordon Setters, Kromfohrländers, Border Collies, and Australian Shepherds, and one Standard Poodle, Norwegian Elkhound, Boston Terrier, Czech Mountain Dog, Golden Retriever, Borzoi, Australian Kelpie, Whippet, Pumi, Cavalier King Charles Spaniel, Staffordshire Bull Terrier, Finnish Lapphund, Slovak Cuvac, French Bulldog, Bernese Mountain Dog, Cocker Spaniel, and Bullmastiff. The dogs were grouped into three diet groups based on what diet they had been eating for the previous six months: 80 % or more raw, 80 % or more dry, or mixed diet (Table 1). We define the raw diet as various types of unprocessed and unheated animal meat and by-products, which are high in animal protein and fat and low in carbohydrates. We define the dry diet as various commercially provided ultra-processed and often extruded kibbles, all of which are high in carbohydrates and lower in fat than raw diets. The protein in dry diets often originate from both animal and vegetal sources, and all dry diets have added micronutrients. Neither raw nor dry food nutrient composition or ingredient lists were collected for this study. Mixed diets consisted of dry, raw, home-cooked, and/or canned food. We define home-cooked food as foods cooked at home for either humans or dogs, hence also human food leftovers and/or snacks. The mixed diets (n=9) in this study had the following composition: first dog 60 % dry, 40 % raw; second dog 60 % dry, 25 % raw, 15 % home-cooked (added Zn); 3rd 60 % dry, 30 % raw, 10 % home-cooked (added Zn); 4th 70 % dry, 10 % raw, 10 % home-cooked, 10 % canned; 5th 45 % dry, 45 % raw, 10 % home-cooked; 6th 20 % dry, 10 % raw, 10 % home-cooked, 60 % canned; 7th 50 % dry, 50 % raw; 8th 70 % dry, 30 % home-cooked (added multivitamin); 9th 50 % dry, 50 % raw (added Zn, vitamin A and C, kelp, eggshells). The dogs were also grouped in relation to their consumption of wild game: never (n = 29), 1-6 times/year (n = 15), and monthly, weekly, or daily (n = 6); and of rice: never or 1-6 times/year (n = 22), and weekly or daily (n = 13). Dog owners’ smoking habits were included for Cd analyses. The study protocol was approved by the Animal Experiment Board in Finland (ELLA) (permit number: ESAVI/452/2020). All owners signed a written consent form.

### Hair samples

Dog owners were asked not to wash their dogs with shampoo during the two weeks preceding the visit. The fur was carefully cleaned with A12t Dilutus 80 % ethanol disinfectant on a clean cotton gauze and then cut close to the skin using clean stainless-steel scissors. Hair samples were collected primarily from the neck area (n = 45), and in few cases from the chest (n = 2) or tail (n = 3) area. The distal part of the hair sample was discarded, leaving only the 3 cm of hair that had been closest to the skin. The amount of hair in one sample was approximately one tablespoon or 125 mg. Based on visual classification, each hair sample was classified as having either dark or light hair color. This did not however always correspond to the dog’s coat color as a whole. Hair samples were put in individual paper envelopes and stored in room temperature and were all sent in one batch to Analytical Research Laboratories, Inc. (Phoenix, USA) for analysis of Ca, Mg, P, Na, K, Fe, Cu, Mn, Zn, Se, Cr, Pb, Hg, Cd, As, Al, and Ni content. At the laboratory, 40 mg of unwashed hair was digested in PTFE reflux tubes containing a combination of nitric acid and perchloric acid in an open vessel and using a hot block/plate. After digestion, each sample was reconstituted to 2 ml using laboratory grade deionized water, and then analyzed with inductively coupled plasma mass spectrometry (Perkin Elmer ICP-MS nexION 2000B). To ensure the accuracy of the results, quality control procedures were implemented, including the use of known controls at the beginning, middle, and end of every batch of hair samples. Any reading that was out of the normally expected range led to retesting of the hair sample. The element concentrations were reported as µg/g.

### Blood samples

Blood was collected from the cephalic vein into Vacuette 1 mL EDTA for complete blood count and 6 mL plain serum tubes for clinical chemistry. All samples were fasting samples. Complete blood cell counts were determined with the ADVIA 2120i Hematology System with multispecies software (Siemens Healthcare Diagnostics) and the cyanmethemoglobin method for hemoglobin measurements. For the biochemical analyses, the collected blood was allowed to clot and then centrifuged (2100× g, 15 min). Measurements were performed using a Konelab 30i chemistry analyzer (ThermoFisher Scientific). Analyses were performed immediately after collection, though in some cases (when samples were taken in Pohjanmaa), samples were sent by mail and analyzed the following day.

For multielemental analysis, blood was collected from the cephalic vein into 6 ml NH Trace Elements Sodium Heparin tubes and then divided into 1.5 ml Eppendorf tubes and stored in -20 °C until their analysis 6-12 months later. All samples were fasting samples. Analysis of whole blood Se, Zn, Cu, Mn, Fe, Cr, As, Cd, Hg, and Pb was performed at the Department of Environmental Sciences, Jožef Stefan Institute (Ljubljana, Slovenia). Altogether 0.3 g of whole blood samples was transferred into pre-cleaned teflon digestion vials. Samples were digested with 0.5 ml of 65 % nitric acid (suprapur) in a microwave system (ULTRAWAVE, Single Reaction Chamber Microwave Digestion System, MILESTONE, Italy) using the following protocol: (1) 20 min temperature rise to 240 °C, (2) kept 12 min at 240 °C and max 100 bar. Digested solutions were transferred into measuring tubes and diluted to 5 ml with Milli-Q water. Prepared solutions were measured by Triple Quadrupole Inductively coupled plasma Mass spectrometry (ICP-QQQ, Agilent 8800, California, USA). Isotopes monitored were: ^52^Cr, ^55^Mn, ^57^Fe, ^63^Cu, ^66^Zn, ^75^As, ^78^Se, ^114^Cd, ^202^Hg, and ^208^Pb. External calibration was used for quantification. Accuracy of results was checked by the use of two reference materials: Seronorm Whole blood Level 1 (lot: 1,702,821) and Level 2 (lot: 1,702,825). The quality control results are presented in Online Resource 1. Limits of detection for blood elements used were three times the standard deviation of several blank samples: 1.5 ng/g for Cr, 0.4 ng/g for Mn, 40 ng/g for Fe, 1.5 ng/g for Cu, 150 ng/g for Zn, 0.2 ng/g for As, 1 ng/g for Se, 0.02 ng/g for Cd, 0.04 ng/g for Hg and 0.4 ng/g for Pb. The study results are reported as ng/g.

### Statistical analyses

SPSS software (version 25; IBM SPSS Statistics) was used for all statistical analyses. The normality of data was assessed using the Shapiro-Wilk test and a natural log- or square root transformation was applied on element variables that did not follow a normal distribution (16/17 hair elements, 6/10 blood elements). Concentrations below the limit of detection (LOD) were assigned a value of LOD divided by the square root of 2. Correlations between hair and blood elements were assessed using Pearson’s correlation. General linear models (GLM) were used to determine the effect of diet (raw, dry, or mixed), sex (male or female), hair color (light or dark), and age on hair and blood element concentrations. In addition, the effect of consuming Pb-shot game (never, 1-6 times per year, or monthly/weekly/daily) on hair and blood Pb concentration, and the effect of consuming rice (never or weekly/daily) on hair and blood As concentration was also assessed. Main effects of diet, sex, hair color, and age on element concentrations, as well as possible interactions (one by one) were tested, and non-significant terms were dropped from the final models. The assumption of error variance equality was assessed using Levene’s test and the Bonferroni correction was used for pairwise comparisons between diet groups. Blood Cd had a large number of <LOD values (21/46, 45.7 %) and hair As a large number of dogs (32/46, 69.6 %) showing the lowest value reported by the laboratory analysis results (0.01 µg/g). In these cases, the Mann Whitney U/Kruskal-Wallis ANOVA test was performed to assess differences between subgroups. Statistical significance was set at P < 0.05 in all analyses.

## Results

### Study population

Clinically healthy dogs (n = 50) were included in this study. The characteristics of the study population are presented in Table 1.

**Table 1:** Characteristics of the study population

Signalment		Study population (N=50)
Mean age (min-max), years		4.5 (1.0-12.1)
Sex, n (%)	Male	25 (50)
	Female	25 (50)
Hair color, n (%)	Light^a^	29 (58)
	Dark^b^	19 (38)
	Mixed^c^	2 (4)
Diet, n (%)	Raw^d^	21 (42.9)
	Dry^d^	19 (38.8)
	Mixed^e^	9 (18.4)

^a^70-100 % of hair sample was white, cream, red, or gray

^b^70-100 % of hair sample was black or brown

^c^50 % of hair sample was black and 50 % red (excluded from statistical analyses involving hair color)

^d^80 % or more of total diet for a minimum of six months

^e^Mix of dry, raw, home-cooked and/or canned food for a minimum of six months (one dog had only eaten the current diet for 1.4 months and was therefore excluded)

### Hair and blood analyses

Mean hair element concentrations are presented in Table 2. The following extreme outliers (±3 times the interquartile range) were removed among hair elements and are mentioned in the discussion where appropriate: Ca (3560 and 2470 µg/g); Na (10,460 µg/g); K (730 and 730 µg/g); Fe (375 and 138 µg/g); Mn (10.35, 5.08, and 2.6 µg/g); Cr (2.25 µg/g); Pb (0.41 µg/g); Hg (0.55 and 0.27 µg/g); Cd (0.04, 0.02, 0.02, and 0.02 µg/g); Al (403.5 and 105.4 µg/g); As (0.22, 0.22, 0.15, and 0.14 µg/g); Ni (6.23, 1.67, and 1.39 µg/g).

**Table 2:** Hair element concentrations (µg/g) in clinically healthy dogs (N=50)

Element concentrations from literature (So et al. 2016)	Group	n	Mean	SD	Min	Max
Ca 588.00± 307.85 (mean ±SD)	All dogs	48	508.75	383.52	100	1950
	Raw	19	327.37	219.67	130	790
	Dry	19	586.84	361.30	100	1310
	Mixed	9	730.00	560.40	260	1950
Mg 124.00± 66.53	All dogs	50	155.60	126.24	30	590
	Raw	21	135.71	158.13	30	590
	Dry	19	158.42	88.71	30	330
	Mixed	9	197.78	119.56	80	460
P 260.00± 37.71	All dogs	50	311.80	61.90	210	490
	Raw	21	306.19	75.73	210	490
	Dry	19	314.74	38.35	260	410
	Mixed	9	318.89	75.24	230	420
Na 2854.00± 859.25	All dogs	49	1793.88	1127.90	110	5130
	Raw	20	1609.50	982.52	110	3570
	Dry	19	1911.05	1232.45	680	5130
	Mixed	9	1838.89	1289.69	240	3790
K 142.00± 76.85	All dogs	48	167.71	110.50	40	560
	Raw	20	164.50	132.25	40	560
	Dry	19	162.11	97.62	60	490
	Mixed	8	187.50	97.06	40	340
Fe 16.20± 4.94	All dogs	48	33.48	17.50	13	98
	Raw	20	31.20	15.02	13	66
	Dry	19	31.90	17.22	13	69
	Mixed	8	42.00	23.87	27	98
Cu 8.80± 1.03	All dogs	50	8.34	1.35	6	13
	Raw	21	8.76	1.61	6	13
	Dry	19	7.95	0.91	7	10
	Mixed	9	8.33	1.32	6	10
Mn 0.39± 0.17	All dogs	47	0.69	0.44	0.17	2.31
	Raw	20	0.72	0.47	0.17	1.72
	Dry	19	0.58	0.31	0.20	1.37
	Mixed	7	0.90	0.65	0.43	2.31
Zn 136.00± 8.43	All dogs	50	135.60	15.54	100	170
	Raw	21	142.38	16.40	110	170
	Dry	19	131.58	12.14	110	160
	Mixed	9	130.00	15.81	100	150
Se 1.01± 0.19	All dogs	50	0.53	0.24	0.10	1.33
	Raw	21	0.63	0.28	0.24	1.33
	Dry	19	0.45	0.21	0.10	0.95
	Mixed	9	0.45	0.15	0.21	0.74
Cr 1.07± 0.20	All dogs	49	0.88	0.25	0.42	1.73
	Raw	20	0.87	0.27	0.42	1.53
	Dry	19	0.87	0.18	0.53	1.19
	Mixed	9	0.93	0.33	0.61	1.73
Pb 0.06± 0.05	All dogs	49	0.09	0.06	0.01	0.30
	Raw	21	0.09	0.06	0.02	0.23
	Dry	19	0.07	0.07	0.01	0.30
	Mixed	8	0.12	0.04	0.07	0.21
Hg 0.08± 0.03	All dogs	48	0.08	0.04	0.01	0.21
	Raw	21	0.08	0.05	0.01	0.21
	Dry	18	0.07	0.04	0.02	0.14
	Mixed	8	0.09	0.04	0.03	0.14
Cd^a^ 0.020± 0.000	All dogs	46	0.01	0	0.01	0.01
	Raw	20	0.01	0	0.01	0.01
	Dry	17	0.01	0	0.01	0.01
	Mixed	8	0.01	0	0.01	0.01
As 0.09± 0.07	All dogs	46	0.02	0.02	0.01	0.09
	Raw	21	0.02	0.02	0.01	0.09
	Dry	17	0.02	0.02	0.01	0.08
	Mixed	7	0.02	0.01	0.01	0.04
Al 4.82± 3.27	All dogs	48	21.42	12.93	6.70	63.30
	Raw	21	21.56	12.02	6.70	50.60
	Dry	19	21.59	15.99	6.70	63.30
	Mixed	7	21.36	7.53	15.00	35.70
Ni 0.10± 0.07	All dogs	47	0.24	0.17	0.06	0.90
	Raw	19	0.27	0.20	0.09	0.90
	Dry	19	0.19	0.12	0.06	0.47
	Mixed	8	0.29	0.14	0.09	0.57

Concentrations from literature (So et al. 2016) are reported under the chemical symbol

Extreme outliers excluded; n, number of dogs included for each element; mean, arithmetic mean; SD, standard deviation

^a^All dogs showed the lowest value reported by the laboratory analysis results (0.01 µg/g)

Mean blood element concentrations are presented in Table 3. Three dogs had not fasted prior to blood sampling and were excluded. The following extreme outliers were removed among blood elements and are mentioned in the discussion where appropriate: Zn (8420 and 7160 ng/g); As (11.85 and 6.38 ng/g); Cd (0.13 ng/g); Hg (5.33 ng/g); Pb (77.39 and 48.78 ng/g).

**Table 3:** Blood element concentrations (ng/g) in clinically healthy dogs (N=50)

Element concentrations from literature if found	Group	n (<LOD)	Mean	SD	Min	Max
Fe 116400± 6700 (mean± SE)^a^ 551886.79± 16037.74 (mean± SD)^b^	All dogs	47	605382.98	62129.09	486000	742000
	Raw	20	610400.00	67522.63	486000	742000
	Dry	18	607777.78	50774.65	508000	706000
	Mixed	8	587625.00	79260.76	503000	713000
Cu 480± 40; 290-740 (mean± SE; range)^a^	All dogs	47	475.73	54.02	386.40	614.20
	Raw	20	483.79	59.04	349.70	614.20
	Dry	18	475.58	52.31	386.40	565.20
	Mixed	8	461.48	48.68	413.40	531.70
Mn 55.15; 46.29-64.28 (median; range)^c^	All dogs	47	30.84	9.78	14.50	59.50
	Raw	20	27.52	8.19	14.50	46.70
	Dry	18	33.54	10.50	16.60	59.50
	Mixed	8	33.40	11.00	20.20	48.80
Zn 4760± 170; 3690-5930 (mean± SE; range)^a^	All dogs	45	3685.56	542.69	2850	5630
	Raw	20	3639.00	429.77	2850	4270
	Dry	17	3714.12	679.15	2930	5630
	Mixed	7	3765.71	570.58	3120	4820
Se 320.75; 132.08-943.40 (median; range)^d^	All dogs	47	388.14	40.95	310.10	476.60
	Raw	20	379.58	42.01	320.00	476.60
	Dry	18	398.22	40.04	310.10	457.90
	Mixed	8	389.37	42.45	341.70	453.60
Cr	All dogs	46 (31)	3.67	6.07	1.06	24.83
	Raw	19 (16)	3.03	5.86	1.06	24.83
	Dry	18 (11)	4.17	6.31	1.06	23.49
	Mixed	8 (4)	4.17	7.02	1.06	21.36
Pb 1; 1-4 (median, IQR)^e^	All dogs	45 (2)	3.50	2.72	0.28	12.37
	Raw	18 (1)	3.71	2.98	0.28	11.61
	Dry	18 (1)	3.39	3.00	0.28	12.37
	Mixed	8	3.32	1.73	1.59	5.63
Hg 0.16-12.38 (min-max)^f^	All dogs	46	0.53	0.35	0.12	1.61
	Raw	20	0.52	0.25	0.20	0.90
	Dry	17	0.51	0.40	0.10	1.60
	Mixed	8	0.63	0.45	0.10	1.40
Cd	All dogs	46 (21)	0.03	0.01	0.01	0.06
	Raw	20 (13)	0.02	0.01	0.01	0.05
	Dry	17 (6)	0.03	0.02	0.01	0.06
	Mixed	8 (2)	0.03	0.01	0.01	0.05
As	All dogs	45	1.15	1.09	0.04	4.30
	Raw	20	0.92	0.85	0.04	3.53
	Dry	16	1.07	1.04	0.14	3.62
	Mixed	8	1.98	1.48	0.76	4.30

Concentrations from literature are reported under the chemical symbol if found (e.g., comparable analytical method, healthy dogs)

Extreme outliers and non-fasted cases excluded; n, number of dogs included for each element; LOD, limit of detection; mean, arithmetic mean; SD, standard deviation; IQR, interquartile range; SE, standard error

^a^Panda et al. 2009, has been converted to a comparable unit (µg/g to ng/g)

^b^Bahovschi et al. 2015, has been converted to a comparable unit (mg/l to ng/g)

^c^Ferreira et al. 2017

^d^Viviano and Vanderwielen 2013, has been converted to a comparable unit (µg/ml to ng/g)

^e^Langlois et al. 2017

^f^Sousa et al. 2013

When comparing hair and blood concentrations of individual elements, significant positive correlations were found only for Hg and Pb. All correlations are presented in Table 4.

**Table 4:** Pearson correlations between element concentrations in hair and blood

Element	n	r	p-value
Fe	46	0.083	0.585
Cu	47	-0.049	0.745
Mn	45	0.269	0.074
Zn	45	-0.204	0.179
Se	47	-0.028	0.851
Cr	46	0.068	0.652
Pb	44	0.384**	0.010
Hg	45	0.601***	0.000
Cd		a	a
As	42	0.022	0.889

n, number of dogs included for each element; r, Pearson correlation coefficient

**Correlation is significant at the 0.01 level (2-tailed)

***Correlation is significant at the 0.001 level (2-tailed)

^a^Cannot be computed because hair Cd is constant

### Association of hair elements with dog characteristics and diet

The results of GLMs to assess the effect of sex, hair color, age, and diet on hair element concentrations are summarized in Table 5.

**Table 5:** General linear models (GLMs) relating hair element concentrations with dog characteristics and diet

Hair element		F_df_	p-value
Ca	Model	47.90_3,41_	0.000
	Color	110.82_1_	0.000
	Diet	4.25_2_	0.021
Mg	Model	20.92_5,41_	0.000
	Color	83.84_1_	0.000
	Diet	4.14_2_	0.023
	Sex * color	5.12_1_	0.029
P^a^	Model	4.61_8,38_	0.001
	Sex	9.27_1_	0.004
	Age * color	5.40_1_	0.026
	Diet * color	3.33_2_	0.047
Na	Model	4.96_3,43_	0.005
	Age	6.01_1_	0.018
	Age * color	5.00_1_	0.031
K^a^	Model	5.66_3,42_	0.002
	Color	4.81_1_	0.034
	Age	5.33_1_	0.026
	Age * color	11.56_1_	0.001
Zn	Model	5.48_3,45_	0.003
	Sex	8.56_1_	0.005
	Diet	3.54_2_	0.037
Se	Model	4.72_8,38_	0.000
	Diet	8.73_2_	0.001
	Diet * color	5.62_2_	0.007
	Diet * age	5.95_2_	0.006
Pb	Model	3.67_2,45_	0.033
	Diet	3.67_2_	0.033
Ni^b^	Model	2.88_4,40_	0.035
	Diet * sex	4.43_2_	0.047

Only significant models and factors are shown; F, F-ratio; df, degrees of freedom

^a^The model was significant, but the assumption of equality of error variances could not be met

^b^Due to the mixed diet group only having one male dog, this dog was removed from the analysis

**Fig. 1:**
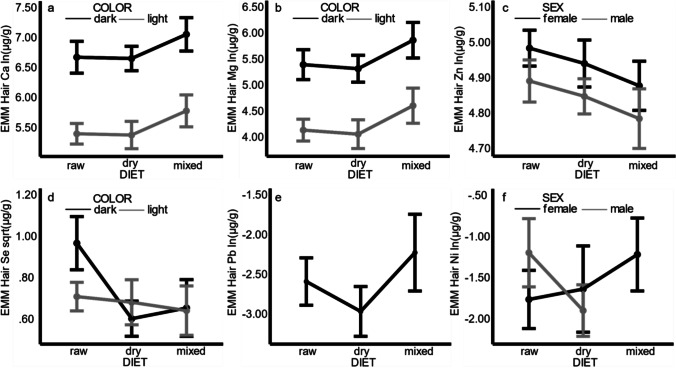
Effect of dog characteristics and diet on hair calcium (**a**), magnesium (**b**), zinc (**c**), selenium (**d**), lead (**e**), and nickel (**f**) concentrations in 50 healthy dogs. EMM, estimated marginal means. The error bars are the confidence intervals for the EMMs

Hair Ca and Mg were significantly higher in dark-colored compared to light-colored dogs, and in dogs that ate mixed diets compared to raw (p = 0.045 for both Ca and Mg) or dry (p = 0.031 and 0.029, respectively) diets (Fig. 1a and b). Also, the effect of hair color on hair Mg concentrations was greater in males than in females, and hair Na increased with age in dark-colored (p = 0.031) dogs.

Hair Zn was significantly higher in female compared to male dogs, and in dogs that ate raw diets compared to mixed diets (p = 0.036) (Fig. 1c). Among the raw diet fed dogs in our study, 11/21 received additional Zn supplements, two ate commercial raw diets with added Zn, and two received a multivitamin that may have contained Zn.

Hair Se was significantly higher in dogs that ate raw diets compared to dry (p = 0.001) or mixed diets (p = 0.005), especially in dark-colored dogs (Fig. 1d). Furthermore, dry diet fed dogs had higher hair Se with older age, mixed diet fed dogs had lower hair Se with older age, and raw diet fed dogs had similar hair Se concentrations at all ages. Of the raw fed dogs, only two received Se-supplemented diets, and two received a multivitamin that may have contained Se.

Hair Pb was significantly higher in dogs that ate mixed diets compared to dry diets (p = 0.039) (Fig. 1e). We could confirm that an extreme outlier for hair Pb among the dry diet fed dogs had an owner that worked at a metal recycling plant. According to the final model, male dogs that ate raw diets had higher hair Ni compared to those that ate dry diets, while female dogs that ate mixed diets had higher hair Ni compared to those that ate raw or dry diets (Fig. 1f).

For hair P and K, we found significant models, but the assumption of equality of error variances could not be met (Levene’s test: F = 2.51, p = 0.021 and F = 4.47, p = 0.04, respectively). Hair P was significantly higher in female compared to male dogs and increased with age in dark-colored dogs. Eating a raw diet had a different effect on hair P depending on the hair color: compared to dogs that ate dry or mixed diets, dark-colored dogs that ate raw diets had higher hair P, and light-colored dogs that ate raw diets had lower hair P. Hair K was higher in dark-colored dogs older than three years of age compared to light-colored dogs and increased with age in dark-colored dogs. For hair Fe, Cu, Mn, Cr, Hg, Cd, As, and Al, we did not find any significant models or factors. Original analysis results for hair element concentrations in individual dogs are presented in separate figures in Online Resource 2.

### Association of blood elements with dog characteristics and diet

The results of GLMs to assess the effect of sex, age, and diet on element concentrations in blood are summarized in Table 6.

**Table 6:** General linear models (GLMs) relating blood element concentrations with dog characteristics and diet

Blood element		F_df_	p-value
Mn	Model	6.72_4,41_	0.000
	Sex	10.14_1_	0.003
	Age	8.78_1_	0.005
	Diet	4.31_2_	0.020
Se	Model	4.47_1,45_	0.040
	Age	4.47_1_	0.040
Pb	Model	6.64_2,42_	0.003
	Age	8.35_1_	0.006
	Pb-shot game	4.91_1_	0.032
As	Model	9.96_2,29_	0.001
	Sex	13.65_1_	0.001
	Rice	6.49_1_	0.016

Only statistically significant models and factors are shown; F, F-ratio; df, degrees of freedom

**Fig. 2:**
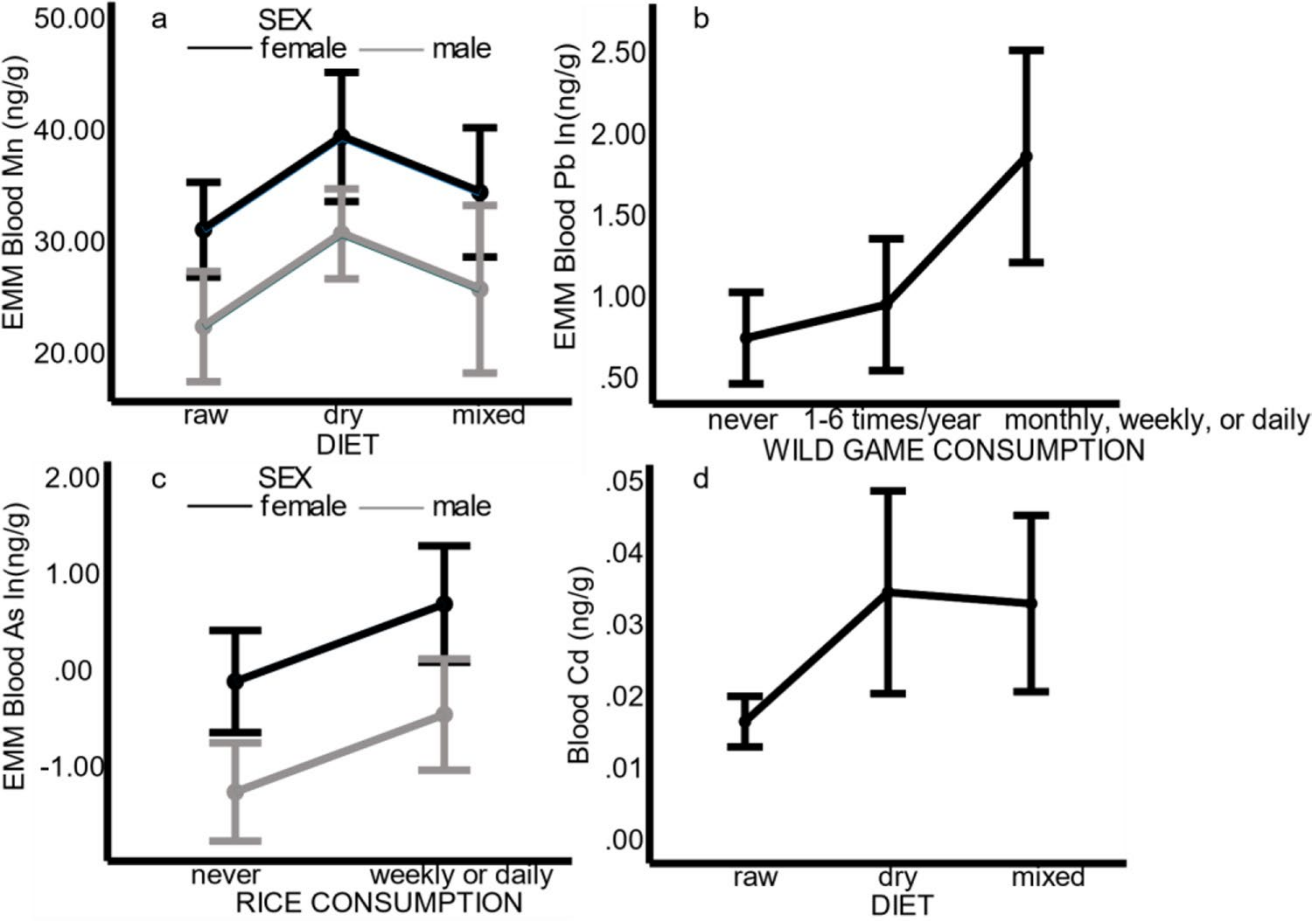
Effect of dog characteristics and diet on blood manganese (**a**), lead (**b**), arsenic (**c**), and cadmium (**d**) concentrations in 50 healthy dogs. EMM, estimated marginal means. The error bars represent the confidence intervals for the EMMs

Blood Mn was significantly higher in female compared to male dogs and increased with age in both sexes. Moreover, blood Mn was significantly higher in dogs that ate dry compared to raw diets (p = 0.017) (Fig. 2a). Blood Se increased with age in all dogs.

Blood Pb decreased with age and was significantly affected by consumption of wild game. Dogs that ate wild game monthly, weekly, or daily had significantly higher blood Pb compared to dogs that never ate wild game (p = 0.009). Dogs that consumed wild game only 1-6 times/year also had higher blood Pb concentrations than those that never consumed wild game, but this difference was not significant (p = 0.063) (Fig. 2b). The mean blood Pb concentration in dogs consuming wild game monthly, weekly, or daily, was 7.2 ng/g (SD 3.14; min 4.36; max 11.61 ng/g), while it was 3.54 ng/g (SD 2.15; min 0.28; max 8.04 ng/g) in those consuming wild game 1-6 times/year, and 2.80 ng/g (SD 2.40; min 0.28; max 12.37) in those that never consumed wild game. Finally, we could confirm that an extreme outlier (12.37 ng/g) among dogs that never consumed wild game, in fact ate a dry dog food containing game meat.

Blood As was significantly higher in female compared to male dogs. Dogs that ate rice weekly or daily had significantly higher blood As compared to dogs that did not eat rice (Fig. 2c). For blood Cd, we found a significant difference between subgroups of diet and dog-owners’ smoking habits (Kruskal-Wallis ANOVA p = 0.028). Post-hoc comparisons revealed that among dogs with non-smoking owners, blood Cd was significantly higher when fed dry (p = 0.003) or mixed diets (p = 0.007), when compared to raw diets (Fig. 2d), whereas there was no significant effect of diet among dogs with smoking owners. However, we did not observe any significant difference in blood Cd concentrations between dogs with smoking and non-smoking owners. No significant models or factors for blood Fe, Cu, Zn, Cr, and Hg were found. Original analysis results for blood element concentrations in individual dogs are presented in separate figures in Online Resource 3.

## Discussion

To determine the correlation between hair and blood element concentrations, and to assess how these elements are affected by diet type and dog characteristics, mean element concentrations in hair and blood were established in healthy companion dogs.

In accordance with previous studies (Tsai et al. 2000; Chyla and Zyrnicki 2000), hair Ca and Mg concentrations were significantly higher in dark-colored compared to light-colored dogs, highlighting the importance of considering hair color in future research. Hair Ca and Mg concentrations were also affected by diet, being higher in mixed diet fed dogs. Hair Ca concentration may reflect dietary Ca content (Ghorbani et al. 2015; Kim et al. 2016), and as many of the mixed diet fed dogs ate complete dry foods mixed with bone-containing raw foods, the total Ca content of their diets was probably higher. However, other dietary factors such as vitamin D and Mg can also raise hair Ca concentrations (Jeruszka-Bielak and Brzozowska 2011). In fact, we removed two extreme outliers for hair Ca, and both dogs’ diets were supplemented with Mg.

In accordance with human studies (Tamburo et al. 2016; Zhu et al. 2018), hair Zn concentration was higher in female compared to male dogs. However, previous dog studies did not find any sex-associated difference in hair Zn (Davies et al. 2017b; Sgorlon et al. 2019). Hair Zn was also higher in raw compared to mixed diet fed dogs. This could be due to either differences in Zn content or in Zn absorption of the consumed diets. The absorption of Zn can be lowered by high dietary amounts of Ca or phytate. Phytate is a substance commonly found in dry dog food ingredients such as corn, wheat, rice, and soy, but rarely in raw, meat based diets (National Research Council 2006). Considering that the mixed diet fed dogs in our study ate diets consisting of 20-70 % dry food, and had higher hair Ca, it is possible that Ca and phytate affected Zn absorption in these dogs. Furthermore, several studies have found higher hair Zn in dogs that are fed diets with organic Zn, as it has better bioavailability compared to inorganic forms (Lowe et al. 1994; Trevizan et al. 2013). The raw fed dogs’ diets included organic Zn from raw ingredients such as red meat, liver, poultry, eggs, and fish (Cummings and Kovacic 2009), whereas dry dog foods commonly include inorganic forms of Zn (National Research Council 2006). The inorganic forms of Zn are also more sensitive to dietary Ca and phytate (Cummings and Kovacic 2009). According to a recent study, only 58.2 % of the total Zn content in dry dog foods was bioavailable to dogs (Gregório et al. 2020). Anyway, the content of Ca, phytate, and Zn in the dogs’ diets were not assessed in this study, and thus, further research is needed to explain the reason for higher hair Zn concentration in raw diet fed dogs. We could not see any difference in hair Zn between the raw diet fed dogs that received Zn supplements and those that did not (data not shown). This suggests that the organic forms of Zn in these meat-based diets is enough to meet the animal’s Zn requirement, since animal-based proteins and the protein-content in the diet is positively correlated with Zn intake (Sandstrom and Cederblad 1980; Sandstrom et al. 1980; Wapnir 2000). The effect of the diet type on the dietary requirements of Zn should be further studied to assure that the requirements are sufficient, and not too high, for dogs that get Zn mostly in organic form.

Blood Mn, which is considered a valid indicator of Mn status in animals (Clegg et al. 1986), was lower in raw compared to dry diet fed dogs. Foods rich in Mn include cereal grains, rice, legumes, nuts, seeds, spinach, seafood, and certain spices such as ginger; while most animal sources, with the exception of feathers (Abduljaleel et al. 2012), wool (Grace 1983), and tripe of grazing animals (Grace 1983; Grace et al. 2008), have very low Mn content (Martins et al. 2020). According to Dillitzer et al. (2011), 10-25 % of raw food diets contain very little Mn, and it is thus possible that some raw fed dogs in our study received too little Mn, especially if the diets did not include tripe or other Mn-rich food sources. In contrast, dry dog foods, which include both Mn-rich ingredients and supplemental Mn (National Research Council 2006), often contain an excess of Mn (Gagne et al. 2013; Pereira et al. 2018; Kazimierska et al. 2020), which could also have impacted on the observed difference in blood Mn concentration between raw and dry diet fed dogs in our study. Due to the lack of data on blood Mn concentrations in healthy dogs, it is difficult to draw conclusions from our results. In a study by Ferreira et al. (2017) healthy dogs fed a standardized dry food had a median blood Mn concentration that was two-fold the mean Mn concentration of the raw diet fed dogs in our study, however the Mn concentrations in their study were overall higher than in ours, so the results might not be comparable. Interestingly, the Mn content in the hair of mixed and raw diet fed dogs seemed to be higher than that of dry diet fed dogs in our study, although not significantly. Further research regarding the effect of diet on blood and hair Mn concentrations in healthy dogs should be conducted in the future. Until then, we recommend ensuring a sufficient Mn content in meat-based raw food diets. Blood Mn was also higher in female compared to male dogs, corresponding to what has been observed in humans (Baldwin et al. 1999; Clark et al. 2007), and it also increased with age, which was previously seen in dogs with non-hepatic illnesses, but not in healthy control dogs (Gow et al. 2010).

Hair Se concentration has been used to indicate dietary Se intake (Górski et al. 2018; Son et al. 2018) and Se status in animals (Christodoulopoulos et al. 2003; Davis et al. 2014). Raw fed dogs had higher hair Se compared to dry and mixed diet fed dogs, suggesting that raw food diets had a higher content or bioavailability of Se. All raw fed dogs were eating animal products of Finnish origin, which may have improved their Se status, considering that Se-fortified fertilizers used in Finland since 1985 have led to a considerably improved Se status of the Finnish population (Pietinen et al. 2010). Whole food ingredients such as meat, offal, fish, or eggs in raw food diets provide Se in organic form (National Research Council 2006), which is more bioavailable compared to the inorganic forms of Se (Moreda-Piñeiro et al. 2017) that are often added to dry dog foods (National Research Council 2006). For example, pigs fed a diet with organic Se had higher hair Se concentration compared to those being fed a similar diet with inorganic Se (Kim and Mahan 2001). Moreover, Se accessibility in pet foods can be negatively affected by heat-processing (Van Zelst et al. 2015). Interestingly, Se has been reported to have a role in the prevention of Ca oxalate calculi in dogs (Santhosh Kumar and Selvam 2003; Liu et al. 2015), as has the raw food diet (Dijcker et al. 2012). The positive effect of raw diets on hair Se concentration was more pronounced in dark-colored dogs, which is in accordance with findings by Kim & Mahan (2001) that dark-haired pigs retained more Se in their hair as dietary Se content increased. Christodoulopoulos et al. (2003) suggested that the phenomenon is caused by a higher content of melanin, containing sulfur amino acids that bind Se. Se increased with age in the blood of all dogs, and in the hair of dry diet fed dogs, but not in the hair of raw or mixed diet fed dogs. In horses, Brummer-Holder et al. (2020) found that blood Se increased with age, while hair Se instead tended to decreased with age, which is similar to what we observed in the raw and mixed diet fed dogs in our study.

Hg concentrations were positively correlated between hair and blood, which is in accordance to findings by Sousa et al. (2013) and Lieske et al. (2011). Pb concentrations were also positively correlated between hair and blood, which has previously been seen in cattle (Patra et al. 2007) and humans (Sanna et al. 2003), but not in dogs. This suggests that hair can be used as a surrogate for blood in assessing dogs’ exposure to Hg and Pb. An extreme outlier for Hg (blood Hg 10-fold and hair Hg 7-fold higher than means) was fed a dry dog food containing 18 % white deep-sea fish. As pet foods containing swordfish, shark, tuna, trout, pike, and bass are known sources of Hg exposure in dogs (Tegzes 2013), we believe that further studies should asses Hg concentrations in dogs that eat these types of fish-based dry foods on a daily basis.

Blood Pb concentration was significantly higher in dogs consuming wild game monthly, weekly, or daily. According to a report by Høgåsen et al. (2016), feeding trimmings of Pb-shot game represent a risk of Pb intoxication in dogs. Eating wild game has also been associated with elevated blood Pb levels in humans (Iqbal et al. 2009), but the interpretation of such studies are complicated by confounding factors such handling of ammunition and inhaling gunfire fumes (Fustinoni et al. 2017; Green and Pain 2019). Thus, dogs who are not prone to these factors, can act as valuable sentinels (Backer et al. 2001). Although the blood Pb concentrations in our study were far below what is considered indicative of Pb toxicosis in dogs (>300-350 ng/g), and no dogs showed signs of gastrointestinal or neurological symptoms associated with Pb toxicity, Pb has no beneficial biological function and the ideal blood Pb concentration is zero (Wismer 2013). Actually, even low-level Pb exposure has been associated with subclinical effects on immune system, organ function, and cognition (Langlois et al. 2017).

Overall, blood Pb concentration increased with age in our study, which is in agreement with previous reports of younger dogs showing higher blood Pb concentrations than older dogs (Lopez-Alonso et al. 2007; Langlois et al. 2017). Moreover, Pb concentrations were significantly higher in dogs eating mixed compared to dry diets, which could possibly be related to a lower nutrient content of these diets (e.g. Zn) leading to an increased absorption of Pb (Wismer 2013). According to Pedrinelli et al. (2017, 2019) micronutrient deficiency is commonly seen in home-cooked diets for dogs. Another possible explanation, considering that Pb accumulates in bone tissue (Fox 1987), is a higher intake of bone and bonemeal in mixed diet fed dogs. This theory is also supported by the higher hair Ca concentration in mixed diet fed dogs. Finally, we removed an extreme outlier for hair Pb, which was probably related to its owner working at a metal recycling plant. It is well known that family members with occupational exposure can bring Pb dust home on clothes and shoes, exposing children to Pb (UNICEF 2020).

Blood As concentration was higher in dogs that were consuming rice daily or weekly compared to dogs that were never consuming rice, however, we did not see any association between rice consumption and hair As concentrations, as we did in our previous study (Rosendahl et al. 2020). The two extreme outliers for blood As were not consuming rice frequently, but both ate fish-based dry foods, which have been associated with higher levels of As compared to other dry foods (Davies et al. 2017a). Unfortunately, due to small sample size, fish consumption could not be assessed in this study, but we recommend that future studies include it as a confounding factor.

In non-smoking people, the main source of Cd exposure is food (European Food Safety Authority 2012). Among dogs with non-smoking owners, blood Cd concentrations were higher in dogs that were fed dry or mixed, compared to raw diets. Tomza-Marciniak et al. (2012) also found that dogs eating commercial or mixed diets had higher serum Cd compared to those that only ate home-made diets. Wheat, rice, and potatoes, all common staple ingredients in dry dog foods, generally contain more Cd than meat, egg, and dairy products (Genchi et al. 2020). Other possible sources of Cd in commercial dog foods are Ca and Zn supplements (National Research Council 2005). Anyway, the observed Cd concentrations in our study were not indicative of toxicity, and the Cd concentration in commercially available dry dog foods (Duran et al. 2010; Abd-Elhakim et al. 2016; Davies et al. 2017b) appears to be well below the legal maximum (European commission 2013). However, even low-level exposure to Cd has been associated with increased oxidative stress (Lovásová et al. 2013) and negative effects on bone health (Åkesson et al. 2014).

Male dogs that ate raw diets had higher hair Ni compared to male dogs that ate dry diets, while female dogs that ate mixed diets had higher hair Ni compared to female dogs that ate raw or dry diets. Due to lack of previous research on hair Ni concentrations in dogs, these findings are difficult to explain and require further research.

Our study has some limitations. The small sample size may have interfered with identifying significant relationships from the data. In addition, to avoid too small sub-groups in statistical models, the number of factors that could be assessed was limited. One excluded factor was the dogs’ living environment, which according to our preliminary data inspection did not have a significant effect on hair and blood element concentrations. We find it more likely that living close to mining or industrialized areas may have affected hair and blood element concentrations in some dogs, and this should be assessed in future studies. We cannot exclude the risk for external contamination of hair samples. According to Chun et al. (2020), acetone-based washing procedures are unsuitable when measuring elements in dogs’ hair because they can alter hair element concentrations, and thus, we instead cleaned the hair with alcohol prior to cutting the hair sample. Furthermore, as we used recently grown hair cut closest to the dogs’ skin, the impact of different hair growth patterns in different breeds of dogs could have affected our results. Another limitation to our study was the broadness of the three diet categories. However, data regarding these broad diet categories may serve as a good base for future studies that may use more specific diets with known macronutrient and ingredient composition, including information regarding the geographical origin of the raw materials. This would further help address and explain the possible role that differing processing methods may have had on the study outcome, especially between raw versus dry food diets. Our study was also limited by the uncontrolled environment and heterogeneity of our study population. Historically studies like this would have been performed using laboratory dogs in a calory cage setting. However, these types of studies are outdated. Besides being unethical, they do not properly mirror the diet and environment of companion dogs and hence do not offer information regarding companion dogs’ exposure to major and trace elements in their natural habitat. Finally, we chose to use whole blood in our study, even though trace elements have previously been assessed in dogs’ serum (Vitale et al. 2019; Cedeño et al. 2020). There are several reasons for this. First, when studying the correlation between hair and blood Hg concentrations, we wanted to be able to compare our results with those by Sousa et al. (2013), who used hair and whole blood to measure Hg in dogs. Second, our study included Pb, which is, according to existing literature, measured from whole blood in both humans (Iqbal et al. 2009) and dogs (Wismer 2013). Third, in the case of certain elements, such as Se, serum is considered to reflect a more short-term dietary intake, whereas whole blood reflects a more long-term intake (Thomson 2004), which we considered more suitable for our study aim. Finally, by using whole blood we minimized the risk of hemolysis of red blood cells interfering with results of intracellular trace elements such as Mn and Fe (Laur et al. 2020).

Nutritional status have been considered a fifth vital sign that should be assessed alongside temperature, pulse, respiration, and pain in the standard physical examination for small animals (Freeman et al. 2011). In this study we presented basic mean values for hair and blood element concentrations in healthy dogs. We further reinforced the evidence that hair can be used as a surrogate for blood in monitoring dogs’ exposure to Hg and Pb. Based on our results, we could also conclude that when compared to dry diets, raw diets do not seem to be associated with lower major or trace element concentrations in dogs, except for in the case of Mn. These findings should be considered in the future, since the recommended allowances for dogs are made mainly for the dry food industry. Since our study dogs were considered healthy, there is no reason to suspect nutrient deficiencies in any of them. Further research needs be conducted to study if there should be different kinds of requirement limits for nutrients in different feeding types. Our findings also suggest that wild game should not be fed frequently to dogs, due to risk for elevated blood Pb concentrations. The significance of lower hair Se and Zn status in dry and mixed diet fed dogs and lower hair Mn status in raw diet fed dogs to dogs’ health requires further research. Finally, we concluded that hair color, age, and sex may affect some hair and blood element concentrations, and therefore these factors need to be considered in future studies. For example, when measuring hair Ca and Mg concentrations in a dog with multiple colors in its coat, we suggest mixing hair of different colors to get a more accurate element status for that dog. Given the scarcity of data on hair and blood element concentrations in dogs, our results are difficult to interpret, and future studies with larger sample size are required to validate our results.

## Supplementary Information

Online Resource 1 (PDF 39 kb)

Online Resource 2 (PDF 156 kb)

Online Resource 3 (PDF 137 kb)

## Data Availability

The datasets generated during and/or analyzed during the current study are available from the corresponding author on reasonable request.
