# Supplementary material for: Diet and dog characteristics affect major and trace elements in hair and blood of healthy dogs
Source: Vet Res Commun. 2021 Nov 6;46(1):261–75. doi: 10.1007/s11259-021-09854-8 (PMC8791866; doi:10.1007/s11259-021-09854-8)
Supplement: Supplementary file 1 — (PDF 39 kb) [file 11259_2021_9854_MOESM1_ESM.pdf]

Diet and dog characteristics affect major and trace elements in hair and blood of healthy dogs

Sarah Rosendahl[1], Johanna Anturaniemi, Kristiina A. Vuori, Robin Moore, Manal Hemida, Anna Hielm-Björkman

[1]Faculty of Veterinary Medicine, Department of Equine and Small Animal Medicine, University of Helsinki, Finland; sarah.rosendahl@helsinki.fi

Table S1 Results obtained for reference materials used for quality control of whole blood element analysis

**Element (ng/g)**	**Seronorm Trace Elements Whole Blood L-1; lot: 1702821**				**Seronorm Trace Elements Whole Blood L-2; lot: 1702825**			
	avg (N=16)	rsd (%)	reference value	reference range	avg (N=16)	rsd (%)	reference value	reference range
Fe	358097	5.4	357000	NA	350956	5.7	350000	NA
Cu	557	5.1	640	590-700	820	6.4	980	890-1060
Mn	17.4	3.9	19.7	18.1-21.3	22.0	7.1	24.2	22.2-26.1
Zn	5483	17.4	4600	3800-5300	6115	5.9	5800	4800-6800
Se	63.3	7.2	69	54-84	125	4.2	144	113-175
Cr	<LOD	NA	0.77	0.61-0.92	9.47	12.0	10.1	8.0-12.0
Pb	9.42	6.9	10	7.9-12	272	4.4	303	272-334
Hg	1.44	14.1	1.57	1.25-1.88	13.9	8.7	16.6	13.3-20.0
Cd	0.32	13.3	0.28	0.23-0.34	5.09	5.5	5.1	4.1-6.1
As	1.92	5.8	2.1	1.7-2.5	10.6	5.8	12.2	9.8-14.7

avg, arithmetic mean; rsd, relative standard deviation; NA, not available; LOD, limit of detection
