# Supplementary material for: Diet and dog characteristics affect major and trace elements in hair and blood of healthy dogs
Source: Vet Res Commun. 2021 Nov 6;46(1):261–75. doi: 10.1007/s11259-021-09854-8 (PMC8791866; doi:10.1007/s11259-021-09854-8)

Diet and dog characteristics affect major and trace elements in hair and blood of healthy dogs

Sarah Rosendahl[1], Johanna Anturaniemi, Kristiina A. Vuori, Robin Moore, Manal Hemida, Anna Hielm-Björkman

[1]Faculty of Veterinary Medicine, Department of Equine and Small Animal Medicine, University of Helsinki, Finland; sarah.rosendahl@helsinki.fi

Fig. S1 Effect of dog characteristics and diet on hair calcium (a), magnesium (b), zinc (c), selenium (d), lead (e), and nickel (f) levels in 50 healthy dogs. Original analysis results for individual dogs

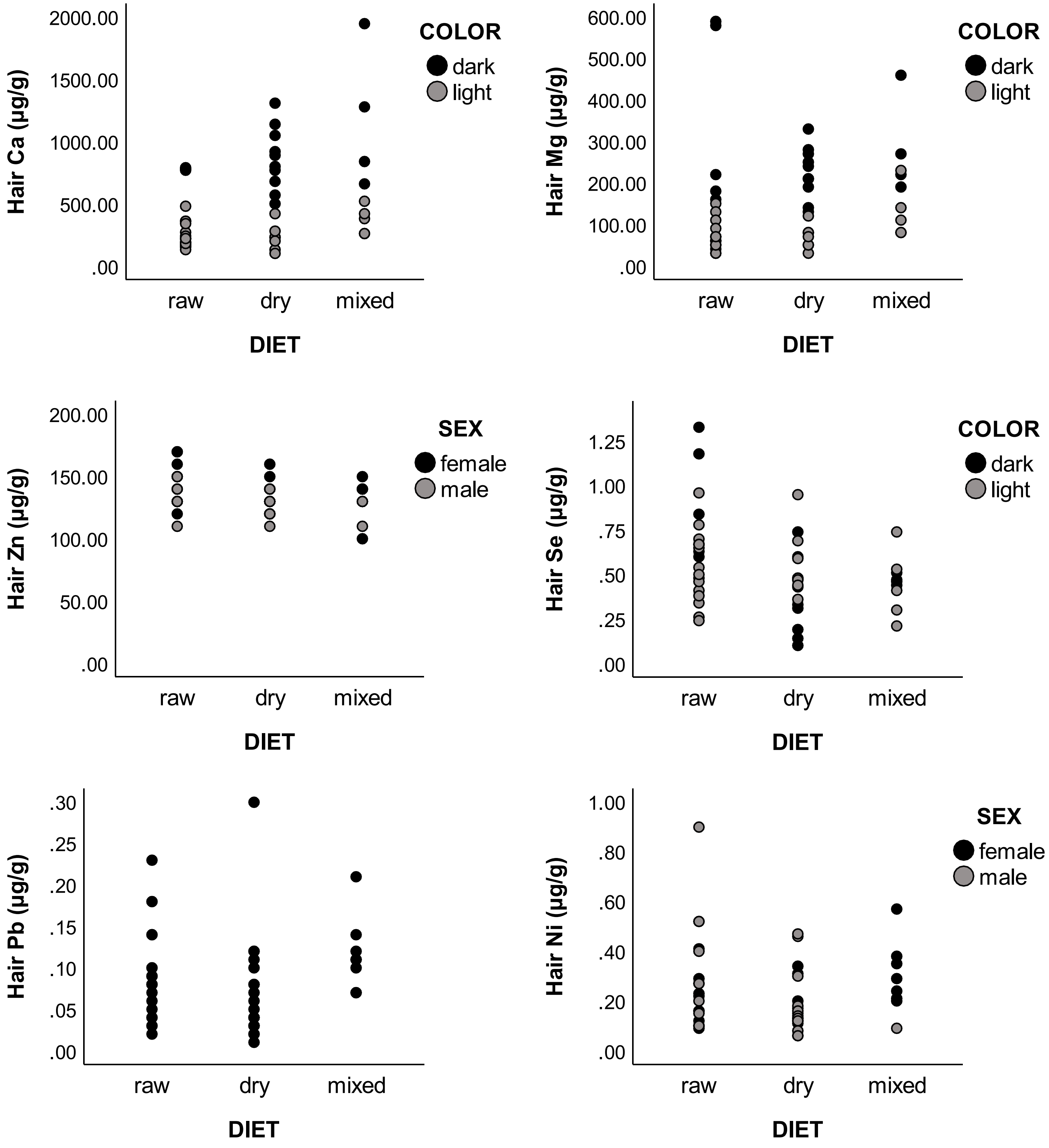

Supplement: Supplementary file 2 — (PDF 156 kb) [file 11259_2021_9854_MOESM2_ESM.pdf]
