# Supplementary material for: Diet and dog characteristics affect major and trace elements in hair and blood of healthy dogs
Source: Vet Res Commun. 2021 Nov 6;46(1):261–75. doi: 10.1007/s11259-021-09854-8 (PMC8791866; doi:10.1007/s11259-021-09854-8)

Diet and dog characteristics affect major and trace elements in hair and blood of healthy dogs

Sarah Rosendahl[1], Johanna Anturaniemi, Kristiina A. Vuori, Robin Moore, Manal Hemida, Anna Hielm-Björkman

[1]Faculty of Veterinary Medicine, Department of Equine and Small Animal Medicine, University of Helsinki, Finland; sarah.rosendahl@helsinki.fi

Fig. S2 Effect of dog characteristics and diet on blood manganese (a), lead (b), arsenic (c), and cadmium (d) levels in 50 healthy dogs. Original analysis results for individual dogs

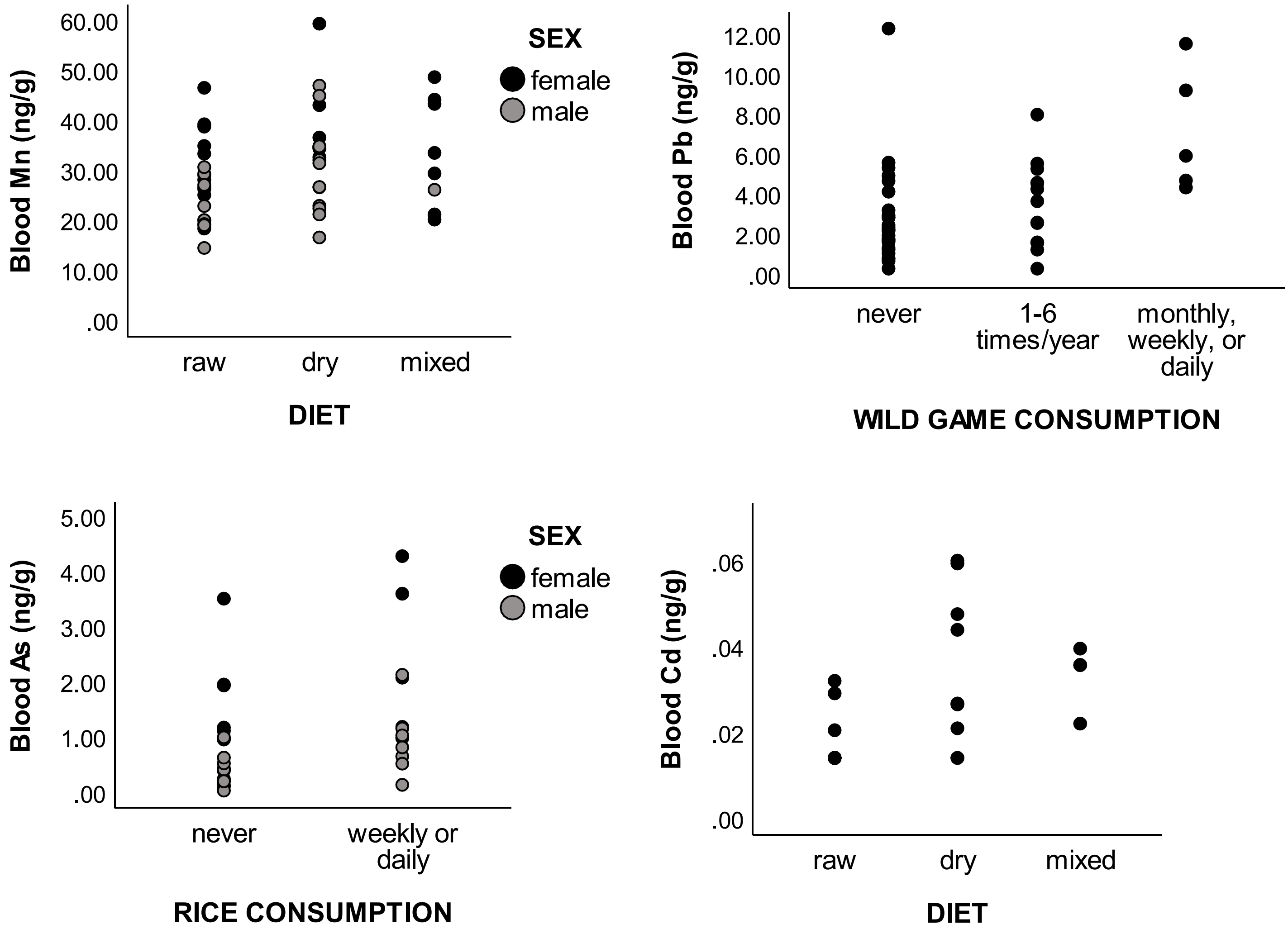

Supplement: Supplementary file 3 — (PDF 137 kb) [file 11259_2021_9854_MOESM3_ESM.pdf]
